# Antimicrobial and anti-inflammatory activities of three chensinin-1 peptides containing mutation of glycine and histidine residues

**DOI:** 10.1038/srep40228

**Published:** 2017-01-05

**Authors:** Weibing Dong, Xiaoman Mao, Yue Guan, Yao Kang, Dejing Shang

**Affiliations:** 1 School of Life Science, Liaoning Normal University, Dalian 116081, China; 2 Liaoning Provincial Key Laboratory of Biotechnology and Drug Discovery, Liaoning Normal University, Dalian 116081, China; 3 State Key Laboratory of Fine Chemicals, Dalian University of Technology, Dalian 116024, China

## Abstract

The natural peptide chensinin-1 doesnot exhibit its desired biological properties. In this study, the mutant MC1-1 was designed by replacing Gly in the chensinin-1 sequence with Trp. Mutants MC1-2 and MC1-3 were designed based on the MC1-1 sequence to investigate the specific role of His residues. The mutated peptides presented α-helicity in a membrane-mimetic environment and exhibited broad-spectrum antimicrobial activities; in contrast to Trp residues, His residues were dispensable for interacting with the cell membrane. The interactions between the mutant peptides and lipopolysaccharide (LPS) facilitated the ingestion of peptides by Gram-negative bacteria. The binding affinities of the peptides were similar, at approximately 10 μM, but ΔH for MC1-2 was −7.3 kcal.mol^−1^, which was 6-9 folds higher than those of MC1-1 and MC1-3, probably due to the conformational changes. All mutant peptides demonstrated the ability to inhibit LPS-induced tumour-necrosis factor-α (TNF-α) and interleukin-6 (IL-6) release from murine RAW264.7 cells. In addition, the representative peptide MC1-1showed better inhibition of serum TNF-α and IL-6 levels compared to polymyxin B (PMB), a potent binder and neutralizer of LPS as positive control in LPS-challenged mice model. These data suggest that the mutant peptides could be promising molecules for development as chensinin-based therapeutic agents against sepsis.

In past decades, conventional antibiotics have been overprescribed, promoting the emergence of multidrug-resistant microbes and a world-wide human health crisis^1^,^2^,^3^. Consequently, the development of antibiotics with novel modes of action has become crucial to combat the problem of resistance^4^. Genome-encoded cationic antimicrobial peptides (AMPs) discovered from bacteria, insects, and vertebratesrepresent an almost inexhaustible source of potential therapeutic agents^5^,^6^,^7^. AMPs serve as a first line of innate immunity and protect the host by exhibiting potent antimicrobial activity against bacteria, fungi and viruses^8^. The mode of action is initiated through electrostatic interactions, causing the adsorption of AMPs at the surface of the negatively charged cell membrane^9^,^10^. The majority of AMPs interfere with membrane permeabilization and membrane-associated enzymes by inserting into the outer membrane hydrophobic core and disrupting the bacterial membrane, leading to cell death^11^,^12^. However, AMPs cannot efficiently bind to the human erythrocyte cell surface due to the presence of zwitterionic lipids on the membrane surface^13^,^14^. In addition to this direct antimicrobial activity, many AMPs often display immunomodulatory activities, such as chemokine induction and endotoxin neutralization to inhibit lipopolysaccharide (LPS)-induced pro-inflammatory cytokine production^15^,^16^. In addition, AMPs also exhibit other biological properties, including the stimulation of angiogenesis, which results in immuno-based anti-infection activity in animal models, and the induction of wound repair^10^,^17^. Taken together, AMPs are the most promising compounds for the development of novel antibiotics^18^.

In our previous work, the 18 amino acid antimicrobial peptide chensinin-1 was purified from the skin secretions of the Chinese brown frog *Rana chensinensis* and characterized^19^,^20^. Three histidine residues are present in the sequence of chensinin-1, which distinguish it from other known antimicrobial peptides produced by amphibians. Histidine typically plays a role of a proton shuttle that can alter antimicrobial activity by adjusting the pH. Chensinin-1 possesses seven positive charges at neutral pH due to the presence of five Arg and two Lys residues; the net positive charge can increase up to +10 under acidic conditions. The N-terminal residues (SAV) in the chensinin-1 sequence differ from those of other antimicrobial peptides with short sequences (i.e., 20–24 amino acid residues) isolated from *Ranidae*. Thus, chensinin-1 is distinct from other known AMPs, including brevinin peptide that contain the ‘Rana box’ domains. In an antimicrobial activity assay, chensinin-1 exhibited potent antimicrobial activity against Gram-positive bacteria, but showed almost no activity against Gram-negative bacteria, which could be due to its relatively low amphipathicity, hydrophobicity, and propensity to primarily adopt a random coil conformation. Chensinin-1 may form oligomers when in contact with the outer membrane, as suggested by the quenching of the fluorescence intensity of rhodamine-labelled chensinin-1 after the addition of LPS. In addition, chensinin-1 decreases LPS-induced production of TNF-α and IL-6, but to a lesser degree than PMS^19^. The novel peptide chensinin-1 could serve as a lead peptide in the design of new anti-infective antibiotics with potent therapeutic applications. Previous research indicated that Trp and Arg residues are not typically present in AMPs in high proportions^21^. The hydrophobic and bulky Trp residue has been reported to facilitate the anchoring of peptides to a bilayer surface via interaction with the interfacial region of the cell membrane^22^. Arg residues can confer positive charge and can form hydrogen bonds as well as cation-π interactions, which render the insertion of Arg residues into a lipid bilayer more favorable^23^. These features unique to Trp and Arg residues make them attractive molecules for use in the design of short-chain antimicrobial peptides. However, the effects of these residues on the overall properties of AMPs remain unclear.

Previous studies indicated that the introduction of Trp residues can significantly improve the antimicrobial activity of the peptide against the Gram-negative bacteria, such as the design of the peptide of IK-6 analogues and chensinin-1b^10^,^24^. In this study, to improve the antimicrobial and anti-inflammatory activities of chensinin-1 and to better understand the specific biology function of His residues in the sequence of chensinin-1, we replaced three Gly residues with Trp to improve the hydrophobicity of the peptide and obtained the novel mutant peptide MC1-1. To investigate the specificity of His residues in the sequence, these residues were removed from the sequence of MC1-1, resulting in peptide MC1-2. Furthermore, we replaced the three His residues with Arg residues to increase the positive charge from +7 to +10; the resulting peptide was called MC1-3. The antimicrobial activity, hemolytic activity, and potential membrane destruction mechanism of the three mutated peptides were examined. The interactions between the peptides and LPS were also determined by dynamic light scattering and ITC experiments in order to better understand the bactericidal activity. Furthermore, the capability of the mutated peptides to block LPS-dependent TNF-α and IL-6 secretion by mouse RAW 264.7 macrophages *in vitro*, as well as mice displaying endotoxemia mice *in vivo* were also investigated.

## Results

### Design and physicochemical properties

To improve the hydrophobicity of the peptide, MC1-1 was designed by replacing Gly residues in the sequence of chensinin-1 with Trp. To investigate the specific function of His residues in the whole sequence, MC1-2 and MC1-3 were designed and synthesized based on the parent peptide MC1-1. For these two mutants, the three His residues were removed from the sequence and/or were replaced with Arg residues, as shown in Table 1. The molecular weight of the peptides was verified by ESI-MS. The theoretically calculated and measured molecular weights of each peptide are summarized in Table 1. The mean hydrophobicity value increased from 4.2889 (chensinin-1) to 9.6888 (MC1-1). After the removal of three His residues, the mean hydrophobicity value was increased to 11.626 (MC1-2); the net charge remained at +7. When the His residues were replaced by Arg residues, the mean hydrophobicity value increased slightly to 9.788 (MC1-3), and the net charge increased to +10. The *H* values of the amino acids used in this study were calculated using reported hydrophobicity scales^25^.

### Secondary structure

**Figure 1:**
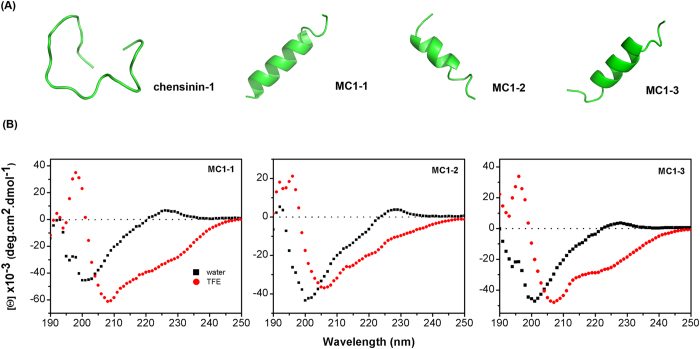
(**A**) Three-dimensional structure simulations of the peptides chensinin-1, MC1-1, MC1-2, MC1-3. (**B**) The CD spectra of the peptides. All the peptides were dissolved in water and 50% trifluoroethanol (TFE) solution.

The secondary structures of MC1-1, MC1-2 and MC1-3 were assessed using CD spectroscopy in water and 50% TFE (trifluoroethanol) solution. Each peptide adopted a random coil structure in water and adopted a significantly ordered α-helical structure in the 50% (v/v) TFE solution, as exhibited by the two local minima at approximately 208 nm and 222 nm (shown in Fig. 1). The calculated percentages of helicity for MC1-1, MC1-2 and MC1-3 (Table 2) were 22.3%, 18.3%, and 15.5%, respectively, in water and 81.2%, 81%, and 80.9%, respectively, in the 50% TFE solution^26^,^27^. The above results were consistent with the predicted results (Fig. 1A and B).

### Antimicrobial and hemolytic activities

The antimicrobial activities of the peptides against the tested bacteria are summarized in Table 3. Compared with the parent peptide chensinin-1, the mutated peptides MC1-1, MC1-2, and MC1-3 demonstrated potent antimicrobial activities against all tested Gram-positive bacteria, with different activities against specific Gram-negative bacteria. To evaluate the overall antimicrobial activity, the geometric mean of the MIC values was calculated^28^. MC1-2 displayed much higher antimicrobial activity against the selected bacteria than MC1-1 and MC1-3. For example, the antimicrobial activity of MC1-2 against Gram-negative bacteria was three-times greater than that of MC1-3 and twice as high as that of MC1-1. Chensinin-1 showed no apparent antimicrobial activity against the selected bacteria, as all measured MICs were over 500 μM. MC1-2 also showed a slightly higher antimicrobial activity against Gram-positive bacteria than MC1-1 and a nearly three-fold increase compared to MC1-3. No peptide showed apparent hemolytic activity against human erythrocytes, HC_50_ > 500 μM (Fig. S1).

### Membrane depolarization

**Figure 2:**
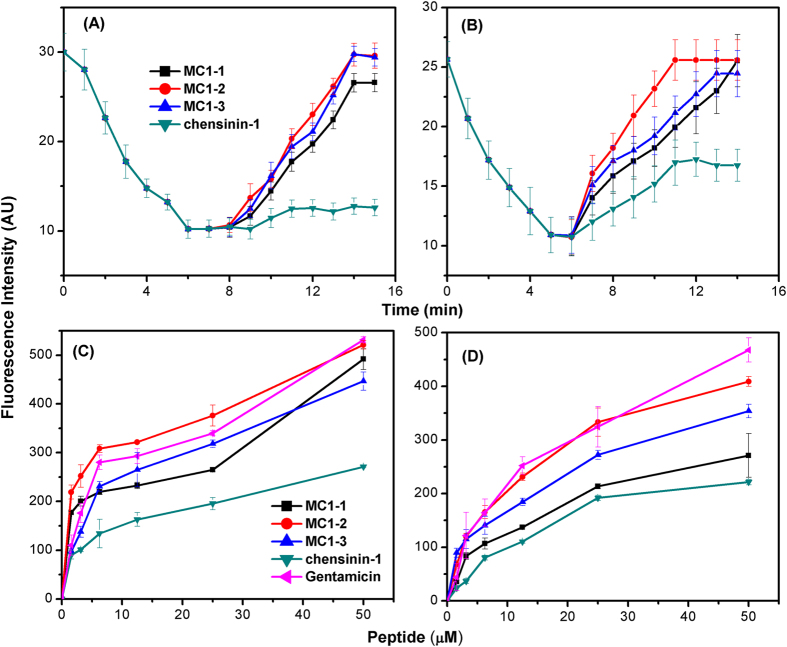
The plasma membrane depolarization of (**A**) *S. aureus* and (**B**) *E. coli* cells by the peptide; The outer membrane permeability of (**C**) *S. aureus* and (**D**) *E. coli* cells induced by the peptide, as determined using the fluorescent dye (NPN) assay.

The dye diSC3-5, which is membrane potential-sensitive, was used to determine the permeabilization ability of the peptides against the intact *E. coli* and *S. aureus* cells membranes. Under the influence of a membrane potential, the dye diSC3-5 is adsorbed onto the cytoplasmic membrane, and its fluorescence is self-quenched. The dye dissociates into the buffer if the membrane potential is disrupted, which causes an increase in the fluorescence intensity. The dye accumulated in the membrane, and the fluorescence intensity of diSC3-5 was significantly quenched (shown in Fig. 2A and B). Once the signal was stable for 1–2 min, the peptide was added, causing a rapid increase in the fluorescence intensity due to the collapse of the ion gradients that induce the membrane potential. All peptides caused the rapid depolarization of the cytoplasmic membrane of *S. aureus* within 7 min. MC1-2 and MC1-3 showed similar depolarization ability, since their respective slope changes in the fluorescence intensity were comparable; both caused rapid depolarization within 6 min. Upon the addition of MC1-2 to *E. coli* cells, complete membrane depolarization occurred in 5 min, as evidenced by a sharp increase in the slope of the fluorescence intensity; but complete membrane depolarization of *E. coli* occurred at 8 min for MC1-1 and MC1-3. This membrane depolarization behaviour was similar to that observed for *S. aureus*. Notably, MC1-2 exhibited the strongest depolarization capacity in both *S. aureus* and *E. coli*, and this result is consistent with the antimicrobial activity of the parent peptide. In comparison, treatment with chensinin-1 resulted in very small changes in the fluorescence intensity, suggesting a lower depolarization capacity compared to the mutated peptides.

### Outer membrane permeability

The permeabilization ability of the peptides with respect to the outer membrane was investigated using the NPN uptake procedure. The hydrophobic fluorescent probe NPN is quenched in aqueous condition, but it shows intense fluorescence intensity in hydrophobic environments. The dye enters the membrane of *E. coli* and *S. aureus* cells when the outer membrane is disturbed, which causes an increase in fluorescence intensity. All peptides induced an fluorescence intensity increase in a concentration-dependent manner with *E. coli* and *S. aureus* cells (shown in Fig. 2C and D), which indicated that these peptides could disrupt the integrity of the outer membrane. In *S. aureus*, MC1-2 caused a sharp increase in the fluorescence intensity. Indeed, the slope of the fluorescence intensity was greater than that of the reference compound gentamicin. In *E. coli*, MC1-2 and gentamicin exhibited similar effects on NPN uptake. Generally, the outer membrane permeabilization induced by MC1-2 was significantly higher than that caused by MC1-1 and MC1-3. Chensinin-1 also demonstrated the ability to induce NPN uptake in *E. coli* and *S. aureus* cells, but its outer membrane permeabilization ability was much lower than that of the mutated peptides. This result is consistent with our previous work^29^.

### Calcein release from liposomes

**Figure 3:**
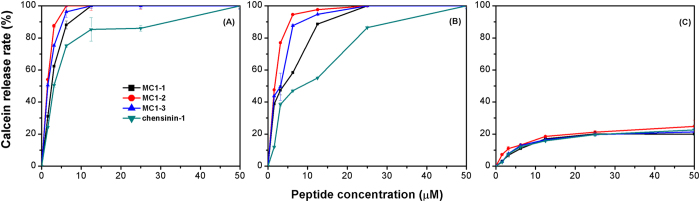
The release of calcein from liposomes composed of (**A**) phosphatidylglycerol (PG) and cardiolipin (CL) (3:1); (**B**) PG, CL and phosphatidylethanolamine (2:7:1); (**C**) phosphatidylcholine and cholesterol (10:1). The fluorescence intensity is known as a function of the time after the addition of a series of concentrations of the tested peptides. The lipid concentration was 50 mM. Each data point represents an average of three independent experiments.

To determine if pores can be formed in the bacterial membrane, membrane disruption was investigated by examining calcein release from three types of liposomes with different compositions. Exposure to the peptides caused the calcein entrapped in the liposomes to leak rapidly in a concentration- and time-dependent manner (shown in Fig. 3 and Fig. S2). MC1-2, at a concentration of 3 μM, induced calcein leakage from PG/CL and PG/CL/PE liposomes of 88% and 77% over 30 min, respectively (red lines in Fig. 3A and B). The leakage induction ability of MC1-2 was stronger than that of MC1-1 and MC1-3. The ability to induce dye release from PG/CL or PG/CL/PE liposomes of the three mutant peptides at 3 μM peptides follows the order of MC1-2 > MC1-3 > MC1-1. When the peptides were added to zwitterionic lipids (i.e., PC/cholesterol), only a small amount of calcein was released from the LUVs (Fig. 3C), which indicated that these peptides cannot disrupt the bilayer of neutral liposomes.

The time courses of calcein leakage following the addition of the mutated peptides were also investigated. In 10 minutes, each peptide (MC1-1, MC1-2, and MC1-3) at a concentration of 20 μM induced 78%, 85%, and 81% leakage, respectively, from PG/CL liposomes and 38%, 42%, and 31%, respectively, from PG/CL/PE liposomes (dark line in Fig. S2). Similarly, 0.5 μM MC1-1, MC1-2 and MC1-3 induced 38%, 47%, and 48% leakage, respectively, from PG/CL liposomes as well as 13%, 16%, and 14% leakage, respectively, from PG/CL/PE liposomes in 10 min (green line in Fig. S2).

The maximum percentage of calcein leakage was achieved following 12 min exposure to each peptide at 20 μM. The peptides were almost ineffective at inducing leakage in uncharged liposomes, as seen in Fig. S2; after 10 min of exposure, only 5% leakage was detected. Completed leakage from the negatively charged liposomes (i.e., an additional 20–40%) was observed upon addition of membrane-disrupting agent Triton X-100 at 12 min.

### Trp fluorescence spectroscopy

**Figure 4:**
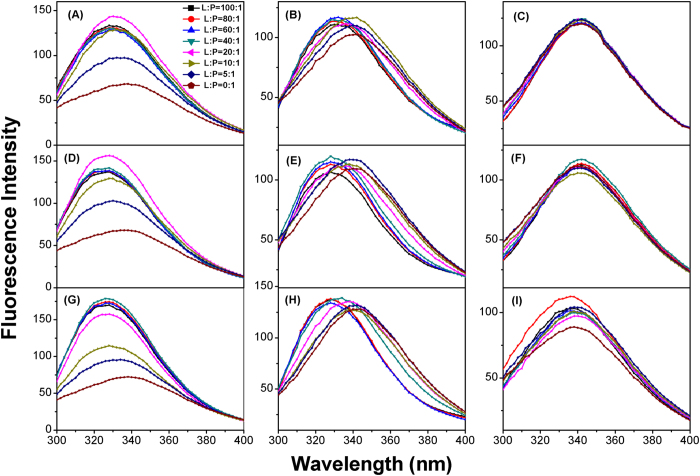
Tryptophan fluorescence emission spectra of the peptides with LUVs model membranes at 25 °C. Fluorescence spectra of MC1-1 in the presence of PG/CL (3:1) (**A**), PG/PE/CL (2:7:1) (**B**) and PC/Ch (10:1) (**C**) liposomes. Fluorescence spectra of MC1-2 in the presence of PG/CL (3:1) (**D**), PG/PE/CL (2:7:1) (**E**) and PC/Ch (10:1) (**F**) lipsomes. Fluorescence spectra of MC1-3 in the presence of PG/CL (3:1) (**G**), PG/PE/CL (2:7:1) (**H**) and PC/Ch (10:1) (**I**) lipsomes.

The Trp residue of the peptide were used as a fluorescent probe to monitor the interactions of the peptides with three types of liposomes: PC/cholesterol (10:1,w/w), PG/CL (3:1,w/w) and PG/CL/PE (2:1:7,w/w/w). The shift in the Trp fluorescence wavelength can be recorded by fluorescence spectroscopy. The peptides exhibited a maximum absorption peak at approximately 340 nm, which indicated that the Trp residues were fully exposed in the aqueous environment (Fig. 4). When negatively charged liposomes were added to the peptide solution, similar blue-shifted wavelength was detected when increasing the lipid concentrations, accompanied by the fluorescence intensity changes in some cases. For MC1-1 and MC1-3, the maximum Trp-fluorescence shifts were 10 nm and 12 nm in the presence of negatively charged PG/CL and PG/CL/PE liposomes; blue shifts were observed at L/P ratios of 5 and 40, respectively (Fig. 4A,G,H and B). The maximum fluorescence shifts of MC1-2 in the presence of the negatively charged PG/CL and PG/CL/PE liposomes were 14 nm and 12 nm, respectively, which were greater than those of MC1-1 and MC1-3, and the L/P ratios were 5 and 40, respectively (Fig. 4D and E). These observations indicated that the peptides can penetrate fully into the negatively charged liposomes. Furthermore, MC1-2 demonstrated much stronger liposome penetration ability, which could be due to the greater hydrophobicity of this peptide, and the insertion into Gram-positive bacteria membranes was greater than that of Gram-negative bacteria membranes. No shifts were detected when the peptides were incubated with neutral liposomes, which suggests that the peptides are unable to interact with the neutral membrane of human erythrocytes (Fig. 4C,F and I).

### Peptide binding to LPS

**Figure 5:**
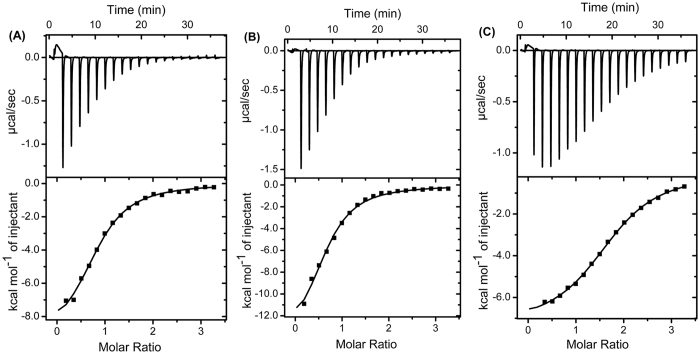
The binding of chensinin-1 analogues with LPS monitored by isothermal calorimetric titration in 10 mM sodium phosphate buffer (pH 6.0) at 37 °C, (**A**) MC1-1, (**B**) MC1-2, and (**C**) MC1-3.

The thermodynamic parameters and binding affinities were determined by ITC and calculated using the one-site binding equation to assess the LPS -peptide interactions (Fig. 5 and Table 4). All interactions between the peptides and LPS were exothermic, as indicated by the downward trend in the ITC profiles. The values for binding affinity, *K*_*d*_, between the peptides and LPS were very close; 10.0 μM, 10.0, μM and 10.5 μM for MC1-1, MC1-2 and MC1-3, respectively. The ΔH and ΔS values of the interaction between MC1-2 and LPS were much greater than those of MC1-1 and MC1-3 (Table 4).

### Disassociation of LPS micelles induced by the peptide

**Figure 6:**
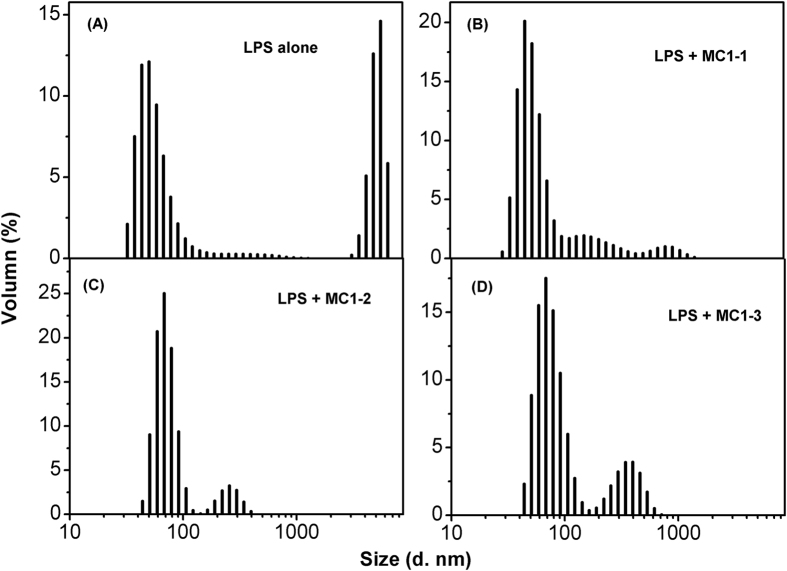
Dissociation of LPS micelles by peptides. Size distribution of LPS micelles in the absence (**A**) and presence of the peptides MC1-1 (**B**), MC1-2 (**C**), and MC1-3 (**D**).

Dynamic-light-scattering experiments were performed to investigate the disassociation of LPS caused by the peptides. LPS alone was initially present in a two sizes distribution with average diameters of 100 and 6000 nm (Fig. 6). The addition of the peptides led to the dissociation of the larger LPS aggregates into smaller-sized aggregates; for the peptides MC1-1, MC1-2 and MC1-3, the most abundant resulting particle sizes were 712 nm, 255 nm, and 396 nm respectively (Fig. 6B,C and D). Additionally, the aggregation centered at 7000 nm disappeared completely. The addition of parent peptide chensinin-1 didnot affect LPS aggregates dissociation, as shown in Fig. S3.

### Surface charge neutralization

**Figure 7:**
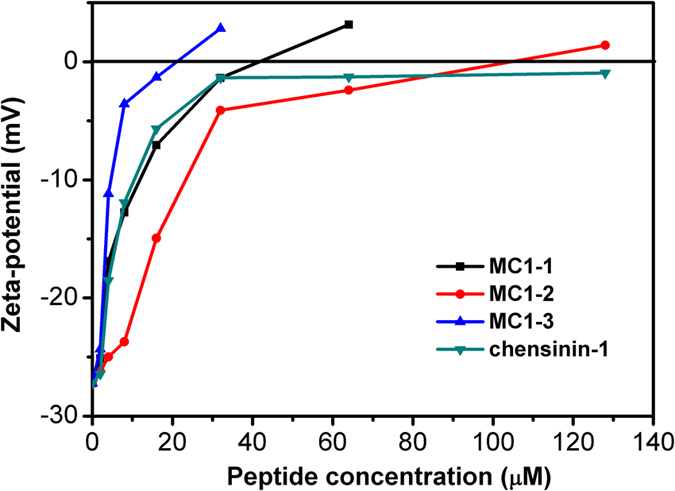
The effect of chensinin-1 mutated peptides on the surface charge of *E. coli* cells.

Zeta potential studies were performed to monitor the effect of the peptides on *E. coli* membrane surface charges. As shown in Fig. 7, the *E. coli* cells showed a zeta potential of −27.2 mV in the absence of the peptides. The addition of the peptides caused a concentration-dependent increase in the zeta potentials. A distinct increase in the zeta potential of *E. coli* cells was observed after the addition of MC1-3; the negative charge at the membrane surface was neutralized at a MC1-3 concentration of 32 μM. A similar trend was observed for MC1-1 and MC1-2, though a greater concentration was needed to fully neutralize the negative surface charge. The reference peptide chensinin-1didnot neutralize the surface charge, even after the concentration reached 128 μM.

### Inhibition of cytokine secretion induced by LPS

**Figure 8:**
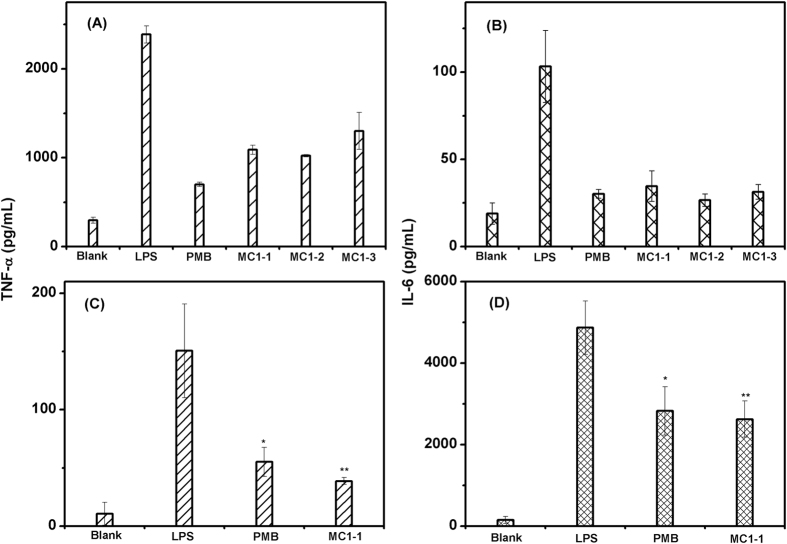
The production of TNF-α and IL-6. Raw 264.7 cells were stimulated with LPS and administrated by MC1-1, MC1-2 and MC1-3 for 24 h (**A** and **B**) and the inhibition of LPS-induced TNF-α and IL-6 release in endotoxemia mice by MC1-1 (**C** and **D**). The concentration of TNF-α and IL-6 was measured by ELISA. The numbers above the bars represent the average inhibition as a result of the peptides treatment and the standard errors. Compared with the LPS-challenged group, significant difference (p < 0.05) is indicated by an asterisk (*), and extremely significant difference (p < 0.01) is indicated by double asterisk (**).

Murine RAW264.7 cells were used to evaluate the function of macrophages *in vitro*. In this system, the peptides displayed extensive binding to *E. coli* LPS, causing pronounced structure transition in LPS/peptide complexes as determined by CD spectra (Fig. S4). Murine RAW264.7 cells were treated with LPS (1 μg/mL) to effect cytokine secretion and were then treated with MC1-1, MC1-2, and MC1-3. RAW264.7 cells with no LPS stimulation secreted a basal level of TNF-α and IL-6 (Fig. 8A and B). However, after stimulation with LPS, an approximate 8-fold increase in the TNF-α and IL-6 protein levels was observed. The production of TNF-α and IL-6 was inhibited with the addition of the peptides. All peptides, at concentrations yielding 80% cell viability, significantly blocked the TNF-α and IL-6 production elicited by LPS. For MC1-1, the TNF-α production was reduced by 54%, and the IL-6 production was reduced by 67%. For MC1-2, there was a 57% reduction in TNF-α and a 74% reduction in IL-6. For MC1-3, there was a 45% reduction in TNF-α and a 69% reduction in IL-6. In contrast, the well-known LPS antagonistPMB, employed as a control experiment, reduced the TNF-a and IL-6 levels by 70% and 71%, respectively. These results indicated that all the peptides showed the ability to inhibit the production of TNF-α and IL-6, and MC1-2 displayed greater inhibition than PMB.

### MC1-1 reduces LPS response *in vivo*

Having demonstrated potential anti-inflammatory properties *in vitro*, MC1-1 was selected as a representative peptide for *in vivo* experimentation. After mice were challenged with a lethal dose of LPS, mouse groups administered with 40, 80 and 160 μg MC1-1 showed 10%, 70%, and 75% survival, respectively.

To determine whether MC1-1 possessed a protective activity *in vivo*, as shown in Fig. 8C and D, the concentrations of plasma TNF-α and IL-6 in LPS-challenged mice were measured at 151 pg/mL and 4885 pg/mL, respectively. In contrast, the concentrations of plasma TNF-α and IL-6 in LPS-challenged mice treated with MC1-1 were 38 pg/mL and 2632 pg/mL, respectively. When measured after a 20 h challenge with LPS, the production of TNF-α was reduced by 75%, and IL-6 production was reduced by 46%. Comparatively, when treated with PMB, the plasma concentrations of TNF-α and IL-6 in mice were 54 pg/mL and 2818 pg/mL; TNF-α production was reduced by approximately 64%, and IL-6 production was reduced by 42%. Thus, the representative peptide MC1-1 displayed greater inhibition compared to PMB.

## Discussion

AMPs, which are less likely than to cause drug resistance, have been shown to induce irreversible membrane disruption by targeting microbial membranes^30^. In this study, three mutated peptides of chensinin-1 were designed to investigate the specific functions of Trp, His and Arg residues in the sequence. The designed peptides exhibited higher hydrophobicity and/or cationicity compared with the parent peptide. The natural antimicrobial peptide chensinin-1 exhibits low antimicrobial activity and low cell selectivity, which could be due to its low hydrophobicity and random coil structural conformation in a membrane-mimetic environment. Trp residues have been reported to anchor peptides to the bilayer surface of cell membrane due to the presence of the bulky aromatic side chain and are able to form hydrogen bonds with dipole moments. These characteristics make Trp residues very promising in the design of new AMPs. In this study, MC1-1 was synthesized by replacing three Gly residues withTrp residues. CD spectroscopy indicated that the α-helical content of MC1-1 increased sharply from 10.5% to 82.6% in 50% TFE solution, and this result was also confirmed by three-dimensional structure prediction. The Trp residues increased the propensity to form helical structure. MC1-1 showed significant antimicrobial activity against both Gram-positive and Gram-negative bacteria compared with chensinin-1. Histidine residues normally act as proton shuttles and are able to alter antimicrobial activity by adjusting the net positive charge through altering the pH. Thus, MC1-2 was designed by removing the three His residues from the sequence of MC1-1. The peptide MC1-3 was designed by replacing these three His residues with Arg residues. MC1-2 exhibited an α-helical conformation, and its antimicrobial activity was enhanced, possibly due to improvements in hydrophobicity. This increased antimicrobial activity could be reflective of the fact that the His residues do not actually play a vital role in the interaction with the cell membrane. MC1-3 also showed an α-helical conformation but exhibited lower antimicrobial activity than MC1-1 and MC1-2. This result suggests that antimicrobial activity depends on a complicated and sensitive balance between various peptide parameters, including net charge, hydrophobicity, amphipathicity, and secondary structure^31^.

The mutated peptides exhibited good cell selectivity and didnot show obvious hemolytic activities at the tested concentrations. Acidic phospholipids, such as phosphatidylglycerol and cardiolipin, abound in the cell membranes of bacteria and serve as the molecular basis of this selectivity^13^,^14^. In addition, some anionic molecules are present in bacterial cell walls, such as LPS in the outer membrane of Gram-negative bacteria and teichoic acids and lipoteichoic acids in the peptidoglycan of Gram-positive bacteria. By contrast, the outer leaflets of human erythrocytes are mainly composed of zwitterionic phosphatidylcholine and sphingomyelin. Therefore, bacteria are more susceptible to the mutated cationic AMPs, but the mutant peptides cannot bind to the cell membrane of human erythrocytes and thus cannot insert into the hydrophobic core to disrupt the integrity of mammalian cell membrane, which would lead to cell death. This advantage renders AMPs distinct from other antibiotic.

Many studies have demonstrated that the cell membrane is the main target for AMPs. The interactions between the mutant peptides and real and biomimetic membranes were investigated. All mutant peptides induced membrane depolarization in intact *S. aureus* and *E. coli* cells. These peptides exhibited different degrees of depolarization for the two tested bacteria. In particular, MC1-2 exhibited potent depolarization of both *S. aureus* and *E. coli*, as the slope of the depolarization process was much bigger than that of the other two peptides. Similar results were observed in an outer membrane permeability assay. MC1-2 treatment caused stronger NPN uptake compared to MC1-1, MC1-3 and the commercial drug gentamicin. These results were also confirmed by an investigation into the interaction between the peptides and liposomes. PG/CL liposomes were used to mimic Gram-positive bacterial cell membranes, and PG/CL/PE liposomes were used to mimic Gram-negative bacterial cell membranes. PC/cholesterol liposomes were used to mimic human erythrocyte cell membranes. All mutant peptides induced dye leakage from the mimetic cell membranes, but the slopes of peptide concentration vs. leakage from PG/CL liposomes were much greater than those of the PG/CL/PE liposomes. A similar trend was observed in the slopes of time course changes. Almost no dye leakage was observed in the case of the PC/cholesterol liposomes. Notably, MC1-2 demonstrated a greater ability than the other two mutant peptides and the reference peptide chensinin-1 to induce dye leakage. The intrinsic fluorescence of Trp was used to monitor the insertion process. It was observed that the blue shift occurred at a lower L/P ratio for the three mutated peptides, which indicated a possible mechanism of pore formation by these peptides, corresponding to the toroidal pore model^32^.

LPS acts as a barrier to prevent peptides from inserting into the inner membrane and is considered the first target for antimicrobial peptides^33^. LPS released from dead bacteria forms oligomers, which can be recognized by innate immune cells and various defence cells to induce the release of pro-inflammatory cytokines^34^. Therefore, the ability of the peptides to neutralize LPS was investigated. From the CD spectra, it was observed that LPS could induce the formation of α-helical peptide conformations, which can be attributed to the LPS/peptide complex. This hypothesis was confirmed by ITC and dynamic light scattering experiments. The direct binding of the peptides to LPS via electrostatic interactions was observed in the ITC experiments. However, MC1-2 showed much lower ΔH and ΔS values, and this atypical result is possibly due to conformational changes in the peptide that subsequently induce changes in hydrogen bonding and hydrophobicity. Indeed, MC1-2 exhibited a higher hydrophobicity value^35^,^36^. The above results can also be reflected in the zeta potential experiment; all the peptides were able to neutralize the negative charge of the *E. coli* cell membrane, and over-compensation of surface charge was observed, which indicated that all the peptides could insert into the cell membrane. These results were also confirmed by size distribution experiments.

Several AMPs have shown the ability to bind LPS efficiently, so AMPs have been pursued as anti-LPS agents^37^. In previous studies, mutated peptides of chensinin-1 have shown affinity towards LPS. Therefore, the capacity of the mutated peptides to neutralize LPS toxicity was investigated. The results showed that the mutated peptides were able to inhibit LPS-induced TNF-α and IL-6 release from murine RAW264.7 macrophage cells. Furthermore, the representative peptide MC1-1 showed the ability to reduce serum TNF-α and IL-6 levels in mice. Specifically, administration with MC1-1 resulted in an increased survival rate of mice with endotoxemia. Compared to PMB, which is a known LPS neutralizer, mutant peptide MC1-1 showed an enhanced ability to inhibit TNF-α and IL-6 production and to protect mice with endotoxemia from death at the same concentration. In addition, the peptide exhibits no hemolytic activity, while PMB possesses known side effects, including nephrotoxicity, neurotoxicity and hemolysis, that limit its clinical use^38^. We conclude that the mutant chensinin-1 peptides are promising neutralizers of LPS with therapeutic potential in the treatment of sepsis.

In summary, the replacement of Gly residues with Trp residues in chensinin-1 greatly enhanced the broad-spectrum antimicrobial activity of MC1-1 compared with the parent peptide chensinin-1 due to increased hydrophobicity and α-helical conformation. The removal of three His residues in the MC1-1 sequence to give the peptide MC1-2 resulted in higher antimicrobial activity than the other two mutant peptides, MC1-1 and MC1-3. For MC1-3, the His residues in MC1-1 were replaced by Arg residues. In general, His residues, which act as proton shuttles, and Arg residues, which increase net cationic charge, were found to have no direct relation to antimicrobial activity of the mutated sequence. A membrane interaction mechanism study suggested that the mutated peptides bind to the outer membrane via an electrostatic interaction and then insert into the membrane, leading to cell death. The therapeutic potential of the mutant peptides in the clinical treatment of LPS-induced sepsis was reflected their ability to bind LPS directly and inhibit LPS-induced TNF-α and IL-6 release both *in vitro* and *in vivo*, promoting the survival rate of mice with endotoxemia. The peptides are non-hemolytic and are attractive molecules for further development as novel chensinin-based antibiotics and therapeutic agents for sepsis.

## Methods

### Peptide

All peptides were synthesized by Karebay Biochem Ltd. (Ningbo, China) using standard Fmoc chemical protocols. The peptides were purified to above 95% homogeneity by reverse-phase high-performance liquid chromatography (RP-HPLC). The mass of the peptides were characterized by using ESI mass spectroscopy (Agilent 6210, USA).

### Antimicrobial activity

Selected bacterial strains were obtained from the China General Microbiological Culture Collection Center. The antimicrobial activities of the peptides against the bacteria, including five Gram-positive bacteria and two negative bacteria: *Staphylococcus aureus* (AS 1.72), *Bacillus cereus* (AS 1.126), *Streptococcus lactis* (AS 1.1690), *Enterococcus faecium* (CGMCC 1.2334), *Enterococcus faecalis* (CGMCC 1.595), *Escherichia coli* (AS 1.349), and *Pseudomonas aeruginosa* (CGMCC 1.860), were measured using the standard broth micro-dilution method as previously described^39^. Briefly, bacteria were inoculated and grown to mid-log phase in fresh LB medium at 37 °C. Each peptide was 2-fold serially diluted with an initial concentration of 200 μM to 1.56 μM for use. Then, 50 μL/well of the peptide solution was added to each well of a sterile 96-well plate. Subsequently, 50 μL/well of inoculation with 10^6^ CFU mL^−1^ were added to each well, the plate was incubated for 18 h at 37 °C. The minimal inhibitory concentration (MIC) was defined as the lowest concentration of the peptide which completely inhibited the growth of bacteria by visual inspection at 600 nm.

### Hemolysis activity

The hemolytic activity of the peptides was assayed by a previously reported standard procedure^40^. Briefly, the erythrocytes were washed three times and resuspended in PBS buffer, and 50 μL of erythrocytes solution were incubated with 50 mL of each diluted peptide for 3 h at 37 °C. Then, intact erythrocytes were centrifuged and the absorbance of the supernatant was measured at 545 nm. Triton X-100 were served as the positive control, and 0.9% (w/v) NaCl was set as the negative control. The HC_50_ was determined by the minimal peptide concentration that induced 50% hemolysis from three independent experiments.

### Bacterial membrane depolarization

The cytoplasmic membrane depolarization activity of three peptides was determined by using the membrane potential-sensitive fluorescent dye, diSC_3-5_ (Sigma-Aldrich) according to the previously described^40^,^41^. Briefly, *E. coli* and *S. aureus* cells were incubated to mid-log phase at 37 °C and harvested by centrifugation, washed three times, and resuspended to an OD_600_ of 0.05 using 5 mM HEPEs buffer (pH 7.2, 20 mM glucose). Subsequently, the diSC3-5 with a final concentration of 4 μM was incubated with the cell suspension until maximal dye uptake was reached by the cells. For *E. coli* cells, EDTA with a final concentration of 0.5 mM was added to the suspension. The fluorescence intensity was detected at an excitation wavelength of 622 nm and an emission wavelength of 670 nm. Then, the peptides at a final concentration of 2 × MICs were added to the bacteria. Membrane disruption was determined by the fluorescence intensity change. The measurements for each dye concentration were repeated three times.

### Outer membrane permeability assays

The outer membrane permeabilization activity of the peptides was determined using the fluorescent dye NPN assay according to the reported procedure^42^,^43^,^44^. Briefly, bacterial cells were grown to mid-log phase in LB buffer, harvested by centrifugation, washed three times and resuspended in PBS buffer with 0.5 OD_600_. 500 μL of the cell suspensions were mixed with 10 μM NPN. Then, the peptides was added to the mixture with increasing the concentration from 0 to 50 μM. The basal fluorescence intensity was recorded at 350 nm (excitation) and 420 nm (emission). The positive control was using gentamicin with concentration ranging from 0 to 50 μM.

### Liposome preparation

Lipids, including Phosphatidylglycerol (PG), Phosphatidylcholine (PC), Phosphatidylethanolamine (PE), cardiolipin (CL) and cholesterol, were obtained from Sigma-Aldrich (Shanghai, China). Three types of liposomes with different lipid ratio were prepared as follows: PC/cholesterol (10:1, w/w) to mimic the human erythrocyte cell membrane; PG/CL (3:1, w/w) to mimic the *S. aureus* membrane; and PG/CL/PE (2:1:7, w/w) to mimic the *E. coli* membrane^45^,^46^,^47^.

For small unilamellar vesicles (SUVs) preparations, lipids were dissolved in chloroform, the organic solvent was dried by rotary evaporation to form a thin film on the sides of the round bottomed flask, then placed under a stream of nitrogen overnight and resuspended with 1 mL of 5 mM TES buffer (pH 7.4). The liposome suspension was subjected to sonicate for two 10-min cycles.

Calcein-entrapped large unilamellar vesicles (LUVs) were prepared according to literature methods^48^,^49^. Lipids at each of the aforementioned ratios were dissolved in chloroform. After vacuum evaporation and drying with a stream of nitrogen overnight, the lipids were resuspended in 60 mM calcein dye buffer solution (100 mM NaCl, 50 mM TES, pH 7.4). Each suspension was ultrasonicated, and subjected to 10 frozen-thaw cycles in liquid nitrogen and then extruded 11 times through a polycarbonate membrane. Untrapped dye was removed by gel filtration on a Sephadex G-50 column.

### Peptide-induced dye leakage assay

Aliquots of the calcein-entrapped liposomes were diluted to a final lipid concentration of 90 μM lipid prior to use. The peptides were added to and incubated with the aliquots for 15 min at final concentrations of 0.5, 2, 5, and 20 μM. The leakage of calcein was detected by measuring the fluorescence intensity at an excitation wavelength of 485 nm and 530 nm (emission). 100% dye release was obtained from the liposomes with the addition of 10% (v/v) Triton X-100 in Tris buffer (20 μL, pH 7.4). The percentage of calcein release was calculated using the equation^50^: dye release (%) = (F_obs_ − F_0_)/(F_100_ − F_0_) × 100%, where F_obs_ is the fluorescence intensity of a peptide liposome/calcein solution, F_0_ is the fluorescence intensity of liposomes, and F_100_ is the fluorescence intensity of the solvent with the addition of Triton X-100.

### Secondary structure analysis

CD spectra of the peptides at a concentration of 0.3 mg/mL were detected on a Bio-Logic MOS-500 spectropolarimeter equipped with a 1 mm path length quartz cell at 25 °C. Spectra were collected at a wavelength ranging from 190 to 250 nmin water or 50% (v/v) trifluoroethanol (TFE, mimicking the hydrophobic environment of the microbial membrane). The acquired CD spectrum in the appropriate solvent was subtracted from the corresponding peptide spectrum. The helical percentage of the peptides were calculated online using DICHROWEB (http://dichroweb.cryst.bbk.ac.uk/)^51^. The predicted secondary structure type for the peptides were examined online using QUARK online (http://zhanglab.ccmb.med.umich.edu/QUARK/).

### Isothermal titration calorimetry

*E. coli* 0111:B4 LPS samples were dissolved in SP buffer (10 mM, pH 6) and vortexed for 15 min prior to use. The ITC binding experiments were performed using an ITC200 (GE) at 310 K with 50 μM LPS samples in the microcalorimetric cell and each 0.8 mM peptide was set in the sample cell. The total titration consisted of 20 injections with an interval of 120 s for all experiments. The peptides were used to investigated binding affinity difference affected by the specific amino acid. The first bad data point was removed for analysis. The ITC data were analyzed with a one-site binding model to determine the equilibrium association constant (*K*_*a*_), binding stoichiometry (*n*), and enthalpy change (ΔH) by using a MicroCal Origin 5.0 package. The entropy change (ΔS) and Gibbs free energy (ΔG) were determined by the equation ΔG = −RTln*K*_*a*_ and ΔS = (ΔH − ΔG)/T, respectively.

### Zeta Potential Measurements

To investigate the capability of neutralizing the negative charge oncell membrane surface, zeta potential were measured by Malvern Nano ZS 90 Instrument (Worcestershire, UK) at 25 °C. The peptides were diluted to 64, 32, 16, 8, 4 and 2 μM using 10 mM PBS buffer (pH 7.4) as the final concentration. 100 μL of each peptide stock solution was added to 900 μL of *E. coli* cells with 0.2 OD_600_. Before measurement, the suspensions were injected into the cells and equilibrated at 25 °C for 2 min.

### Disassociation of LPS

Dynamic light scattering measurements were performed in a Nano ZS90 and used to determine the ability of the peptide to dissociate the LPS micelles.The peptides and buffer solutions were filtered through 0.45 μm filters before starting the experiments. Measurements were carried out after incubation of 1 mM LPS with or without 2 mM peptides. Data were analyzed using the software supplied with the instrument.

### Inhibition of LPS-induced TNF-α and IL-6 production from RAW264.7 cells

The macrophage cell RAW264.7, purchased from KeyGEN BioTECH (Nanjing, China), were cultured in RPMI-1640 (KeyGEN BioTECH) containing 10% FBS (Gibco, Thermo, Shanghai), 80 μg/ml streptomycin and 80 U/mL penicillin (Gibco) at 37 °C and 5% CO_2_ atmosphere. Then the cells were stimulated with LPS (1 μg/ml) and treated with the peptide at the concentration of 0, 20 or 40 μg/mL for 24 h. After another additional 24 h, the supernatants were collected and analyzed by ELISA kits according to the manufacturer’s recommendations (NeoBioscience, Shenzhen, China). The inhibitory effects of chensinin-1 on the production of IL-6 and TNF-α were evaluated for comparation.

### Experimental animals

Male Kunming mice (8 weeks of age, weighing about 19–21 g) were purchased from Dalian Medical University, China. The animals were housed in cages at 22–25 °C with 12-12 light-dark cycle. The mice were fed with a standard laboratory chow diet and tap water for acclimation. All the animal experiments were carried out in accordance with the approved guidelines of the “Principles of Laboratory Animal Care” (NIH publication #85-23, revised 1985) and were approved by the Animal Welfare and Research Ethics Committee of Dalian Medical University (Permit Number: SYXK2004–0029).

### Inhibition of LPS-induced TNF-α and IL-6 production in mice

MC1-1 was selected as representative to evaluate the *in vivo* treatment effect on LPS challenge. The mice were injected with a lethal dose of 8 mg/kg LPS intraperitoneally (ip). Ten minutes after LPS injection, 40, 80 and 160 μg of MC1-1 was injected ip into the mice. Subsequently, the animals were sent back to individual cages to monitor the rate of lethality.

To examine the efficacy of MC1-1 at inhibiting the LPS-induced TNF-α and IL-6 production in mice, 8 mg/kg LPS was injected ip into the mice, followed by the addition of 80 μg MC1-1. The treatment with 1 mg/kg polymyxin B was set as the positive control. The treatment with saline was used as the experimental control. After LPS and/or peptide administration, the mice survival was monitored for 20 h. Then the mice were anesthetized and blood was collected via orbital sinus for TNF-α and IL-6 measurement. The levels of TNF-α and IL-6 were assessed by using mouse TNF-α and IL-6 ELISA kits (NeoBioscience).

### Statistical methods

The experiments in this paper were repeated three times unless otherwise specified, the results were expressed by using the mean ± standard deviations (SD). All data were performed one-way analysis using ANOVA and *P* value < 0.05 was considered statistically significantly.

## Additional Information

**How to cite this article**: Dong, W. *et al*. Antimicrobial and anti-inflammatory activities of three chensinin-1 peptides containing mutation of glycine and histidine residues. *Sci. Rep.* **7**, 40228; doi: 10.1038/srep40228 (2017).

**Publisher's note:** Springer Nature remains neutral with regard to jurisdictional claims in published maps and institutional affiliations.

## Supplementary Material

Supplementary Information

## Figures and Tables

**Table 1:** Amino acid sequences, molecular weights, charge, and hydrophobicity values of the mutations of the parent peptides chensinin-1.

Peptides	Amino acid sequences	Net charge	*H*^a^	Mass calc./obs^b^
Chensinin-1	SAVGRHGRRFGLRKHRKH	7	4.2889	2155.5/2155.8
MC1-1	SAVWRHWRRFWLRKHRKH	7	9.6888	2542.9/2542.8
MC1-2	SAVWRWRRFWLRKRK	7	11.626	2131.5/2131.2
MC1-3	SAVWRRWRRFWLRKRRKR	10	9.788	2600.1/2600

^a^The mean hydrophobicities (H) of the peptides calculated using the hydrophobicity scales were the total hydrophobicity (sum of all residue hydrophobicity indices) divided by the number of residues.

^b^Mass calc./obs. represented calculated molecular masses based on the amino acid sequence determined by Edman degradation and observed molecular masses determined by Maldi-TOF MS.

**Table 2:** MIC of the peptides against the selected bacteria.

Microorganism	MIC(μM)^a^		
	MC1-1	MC1-2	MC1-3
G^−^			
* E. coli*	6.25	3.13	6.25
* P. aeruginosa*	3.13	1.56	6.25
GM (μM)^b^	4.42	2.21	6.25
G^+^			
* S. aureus*	6.25	3.13	6.25
* B. cereus*	3.13	3.13	6.25
* S. lactis*	6.25	6.25	12.5
* E. faecalis*	1.56	1.56	6.25
* E. faecium*	3.13	1.56	6.25
GM (μM)	3.59	2.72	7.18
HC_50_ (μM)	>500		

^a^Minimum inhibitory concentration (MIC) was determined as the lowest concentration of the peptide that inhibited growth. Data are representative of three independent experiments.

^b^The geometric mean (GM) of the peptide MICs against the bacteria was calculated.

**Table 3:** Percentage of α-helix character of the mutated peptides.

	in H_2_O	in TFE
MC1-1	9.92	82.58
MC1-2	2.79	40.97
MC1-3	10.96	54.07

**Table 4:** Thermodynamic parameters of the interactions of the mutated peptides with LPS.

	MC1-1	MC1-2	MC1-3
N	0.83 ± 0.02	1.75 ± 0.01	0.66 ± 0.03
*K*_*a*_ (mM^−1^)	99.8 ± 10	99.8 ± 4.3	96.7 ± 11.7
ΔH (kcal.mol^−1^)	−39.8 ± 1.5	−7.3 ± 0.1	−62.4 ± 3.4
ΔS (kcal.mol^−1^deg^−1^)	−0.0325	−0.0007	−0.106
ΔG (kcal.mol^−1^)	−29.7	−7.1	−29.6
*K*_*d*_ (μM)	10.0 ± 1	10.0 ± 0.4	10.5 ± 1.2
