# Supplementary material for: Antimicrobial and anti-inflammatory activities of three chensinin-1 peptides containing mutation of glycine and histidine residues
Source: Sci Rep. 2017 Jan 5;7:40228. doi: 10.1038/srep40228 (PMC5215317; doi:10.1038/srep40228)
Supplement: Supplementary Information [file srep40228-s1.pdf]

Antimicrobial and anti-inflammatory activities of three chensinin-1 peptides containing mutation of glycine and histidine residues

Weibing Dong[1,2,3], Xiaoman Mao[1], Yue Guan[1], Yao Kang[1], Dejing Shang[1,2,*]
[1]*School of Life Science, Liaoning Normal University, Dalian 116081, China*
[2]*Liaoning Provincial Key Laboratory of Biotechnology and Drug Discovery, Liaoning Normal University, Dalian 116081, China*
[3]*State Key Laboratory of Fine Chemicals, Dalian University of Technology, Dalian 116024, China*

* Corresponding author:
Liaoning Provincial Key Laboratory of Biotechnology and Drug Discovery,
Liaoning Normal University, Dalian 116081, China
Tel & Fax: +86-411-85827071
Email address: djshang@lnnu.edu.cn (D. Shang);

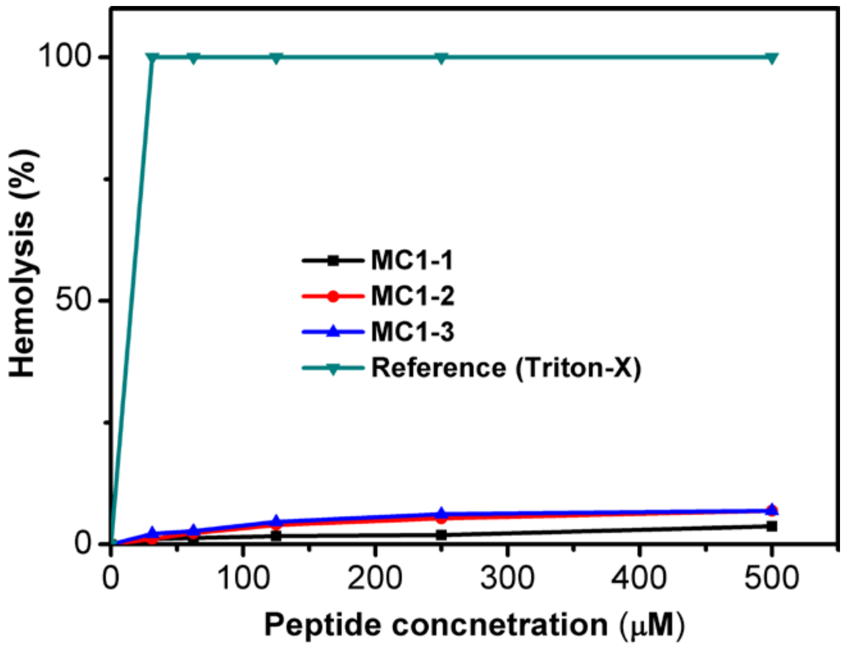

Figure S1. Hemolytic activity of the mutated peptides against human erythrocytes.

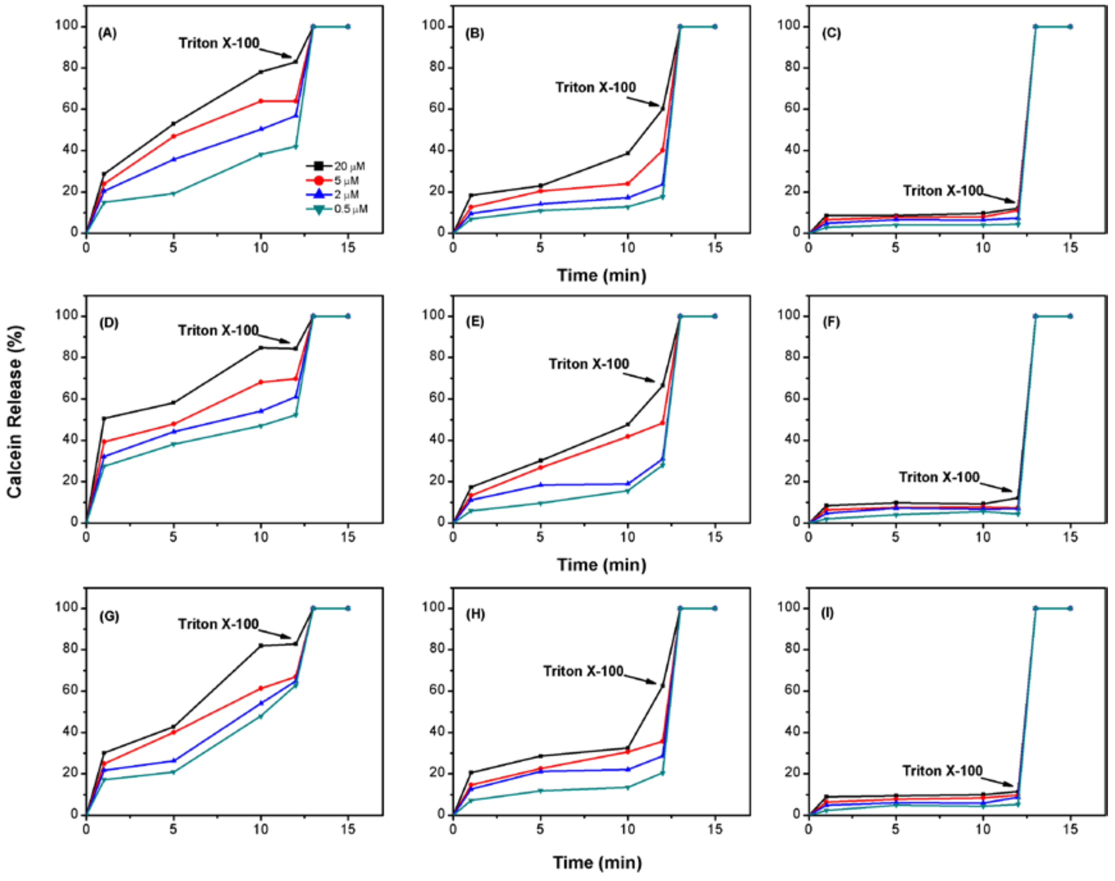

Figure S2. Time courses for the leakage of calcein after the addition of the mutated peptides. The arrow indicates the addition of Triton X-100 at 12 min, causing the complete efflux of calcein from the liposomes. MC1-1 in the presence of PG/CL (3:1) (A), PG/PE/CL (2:7:1) (D) and PC/Ch (10:1) (G) lipsomes. MC1-2 in the presence of PG/CL (3:1) (B), PG/PE/CL (2:7:1) (E) and PC/Ch (10:1) (H) lipsomes. MC1-3 in the presence of PG/CL (3:1) (C), PG/PE/CL (2:7:1) (F) and PC/Ch (10:1) (I) lipsomes.

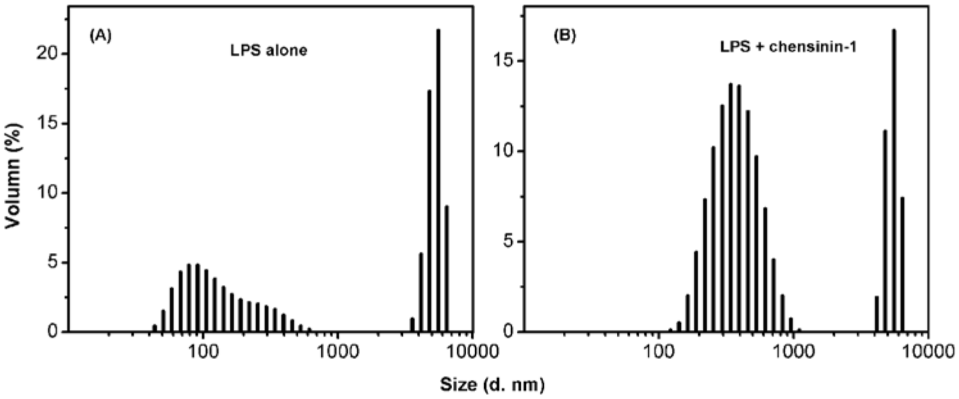

Figure S3. Size distribution of LPS micelles in the absence (A) and presence of the parent peptide chensinin-1 (B).

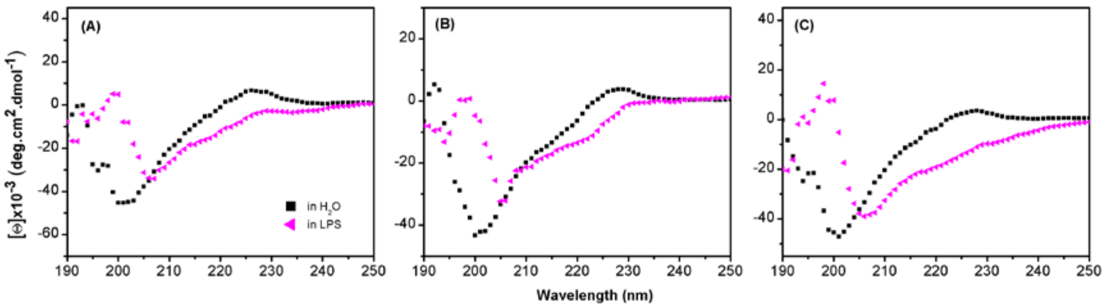

Figure S4. Circular dichroism spectra of the mutated peptides in H_2O and in 50 mM LPS micelles. (A) MC1-1, (B) MC1-2 and (C) MC1-3.
